# BOLD Signal in Both Ipsilateral and Contralateral Retinotopic Cortex Modulates with Perceptual Fading

**DOI:** 10.1371/journal.pone.0009638

**Published:** 2010-03-11

**Authors:** Po-Jang Hsieh, Peter U. Tse

**Affiliations:** Department of Psychological and Brain Sciences, Dartmouth College, Hanover, New Hampshire, United States of America; National Institute of Mental Health, United States of America

## Abstract

Under conditions of visual fixation, perceptual fading occurs when a stationary object, though present in the world and continually casting light upon the retina, vanishes from visual consciousness. The neural correlates of the consciousness of such an object will presumably modulate in activity with the onset and cessation of perceptual fading.

**Method:**

In order to localize the neural correlates of perceptual fading, a green disk that had been individually set to be equiluminant with the orange background, was presented in one of the four visual quadrants; Subjects indicated with a button press whether or not the disk was subjectively visible as it perceptually faded in and out.

**Results:**

Blood oxygen-level dependent (BOLD) signal in V1 and ventral retinotopic areas V2v and V3v decreases when the disk subjectively disappears, and increases when it subjectively reappears. This effect occurs in early visual areas both ipsilaterally and contralaterally to the fading figure. That is, it occurs regardless of whether the fading stimulus is presented inside or outside of the corresponding portion of visual field. In addition, we find that the microsaccade rate rises before and after perceptual transitions from not seeing to seeing the disk, and decreases before perceptual transitions from seeing to not seeing the disk. These BOLD signal changes could be driven by a global process that operates across contralateral and ipsilateral visual cortex or by a confounding factor, such as microsaccade rate.

## Introduction

**Figure 1:**
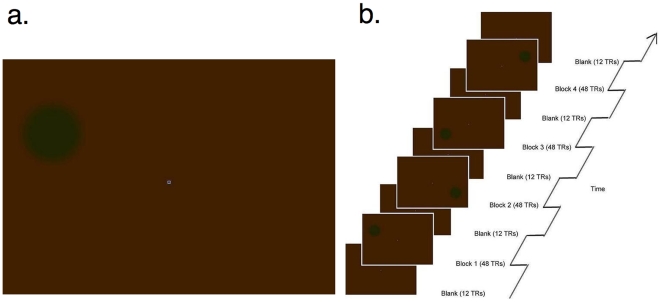
Stimuli. (a) An example stimulus for inducing perceptual fading. After fixating on the central white fixation spot for several seconds, the green disk fades and appears to be replaced by a uniform background. (b) An example run of the experiment.

Perceptual fading, also called “The Troxler effect” [1], occurs when an object, though present in the world and continually casting light upon the retina, vanishes from visual consciousness (**Figure 1a**). Perceptual fading occurs when an object's image is stabilized upon the retina, as when a stationary object is viewed under conditions of careful fixation. Perceptual fading offers a potentially useful tool for localizing the neural correlates of visual consciousness; Areas of the brain where neural activity changes as a function of subjective visibility/invisibility are candidate areas for the neural correlates of visual consciousness.

It is commonly believed that during perceptual fading, the color/texture of the apparently vanished object is filled in with the color/texture of the background because the features of the filled-in area are determined by features located outside the stabilized boundary [2]–. This hypothesis assumes that the filling-in mechanism may be a local process [12], which is based on the local features located at the stabilized boundary. However, we have recently shown psychophysically [13], [14] and using functional magnetic resonance imaging (fMRI) [15] that the filled-in color is not solely determined by the local background color, but is the global mixture of the background and the foreground color. For example, consider a blue disk that is located only in the upper left visual quadrant on a red background that spans the whole visual field; Once this blue disk perceptually fades, the color seen across the entire visual field is an area-weighted average of blue and red, namely, purple. These results suggest that the filling-in mechanism may not simply be a local process, but may involve a more global process of feature analysis across the visual field. Indeed, given past results [13]–[15], we would expect that perceptual fading of a disk located in a single visual quadrant should be evident in both contralateral as well as ipsilateral retinotopic cortex.

Here we used event-related fMRI to examine cortical neural correlates of perceptual fading and subjective reappearance. Our goal was to determine whether perceptual fading is a purely local process or one that also involves global processing. In each stimulation block, an individually equated equiluminant green disk was presented in one of the four quadrants (left top, left bottom, right top, and right bottom) on a dark orange background (**Figure 1**). Observers indicated with a button press whether the disk had undergone perceptual fading or not. Observers typically transitioned back and forth between ‘see’ and ‘no see’ states. We then compared the BOLD signal after perceptual switches by averaging data from ‘see’ conditions that followed ‘no see’ conditions, and vice versa. Since a given retinotopic area (left hemisphere V1v for example) is thought to only respond in a bottom-up manner to a given visual quadrant (right top), we can compare the BOLD signal in different conditions to see whether the BOLD signal only changes when the target disk undergoing perceptual fading is directly located in the area to which a retinotopic area corresponds. For present purposes, we will call this visual area the ‘response field’ of the retinotopic area. If the local hypothesis is correct, the BOLD signal should change only when the target disk is directly located in the response field of the retinotopic area; When the target disk undergoing perceptual fading is not located in the quadrant corresponding to a given retinotopic area, no BOLD signal change should be observed. On the contrary, if the global hypothesis is correct, a BOLD signal change should be observed whether or not the target disk is located in the quadrant corresponding to a given retinotopic area. In particular, if the global hypothesis is correct, we should see ipsilateral as well as contralateral modulation of cortical activity as a function of perceptual fading.

We note that BOLD signal modulations may be due to changes in eye movements such as microsaccade rate and not arise because of perceptual fading per se. To evaluate this possible confounding effect from eye movements, we psychophysically measured the microsaccade rate before and after perceptual transitions to and from perceptual fading.

## Results

### Behavioral Data

**Figure 2:**
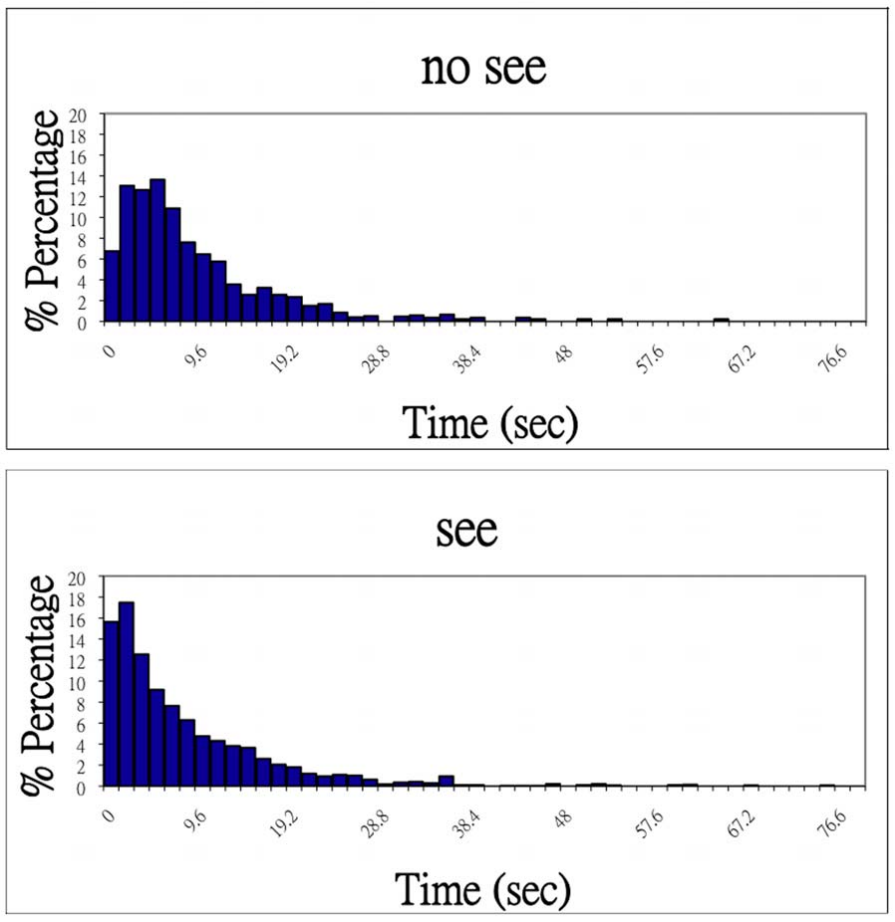
Histograms of perceptual durations (sec). Histograms of perceptual durations (sec) during fMRI experiments accumulated over 6 subjects are shown for the “no see” and “see” percepts.

In the fMRI experiment, the mean perceptual duration (including those percepts shorter than 2 TRs) across 6 subjects for the ‘no see’ state, in which subjects did not see the disk, was 6.47 TRs  = 10.34 sec (median  = 5 TRs  = 8.0 sec; mode  = 4 TRs  = 6.4 sec). The mean perceptual duration for the ‘see’ state, in which subjects saw the disk, was 5.82 TRs  = 9.31 sec (median  = 4 TRs  = 6.4 sec; mode  = 2 TRs  = 3.2 sec) (**Figure 2**).

### Fixation Data during Scanning

Eye movements, wakefulness, and attention to the fixation point were controlled for by requiring subjects to report whether the fixation had changed color by pressing another button with their left index finger. The fixation point changed color randomly from blue/yellow to red/green on average every 3.1 seconds. The results of the fixation task show that the average button-press correct response rate (of the runs that are included in the analysis) after a fixation color change was 84.97%±3.48%, and the average reaction time was 709.11±31.21 ms. Because this color detection task was only possible to carry out while fixating, we can infer that subjects maintained generally good visual fixation.

### Global BOLD Signal Modulations in Early Retinotopic Areas

**Figure 3:**
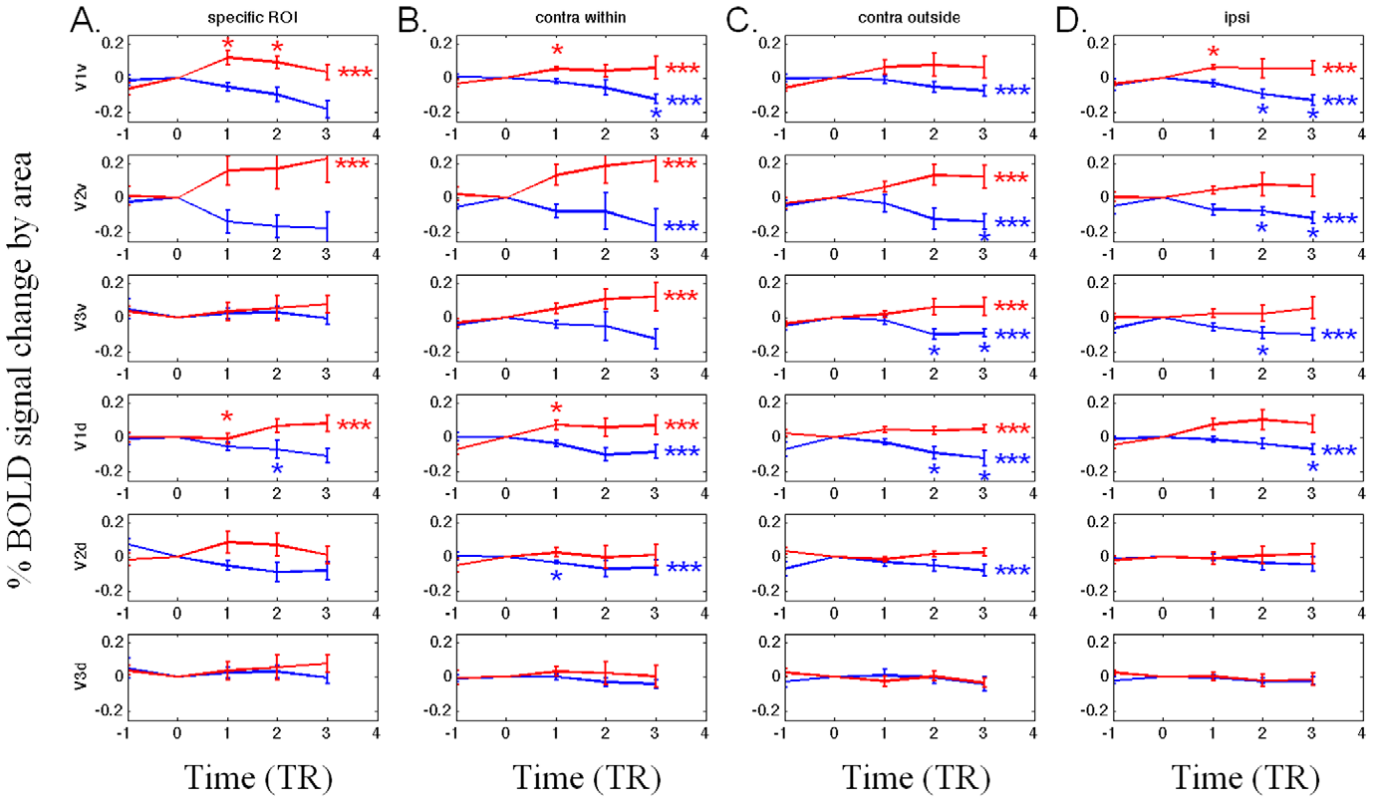
BOLD timecourses. (a) The BOLD signal change averaged across voxels within subjects' ROIs (specific ROIs directly corresponding to the location of the target disk) and averaged across hemispheres and subjects. The BOLD signal was averaged using the subjects' button-press as a trigger, indicated by the ‘0’ point on the horizontal axis. Note that the blue (red) curve indicates the “no see” (“see”) condition after the value x = 0, but indicates the opposite condition at x = -1 because x = 0 corresponds to the moment of a perceptual switch between states. In V1d, V1v, and V2v, the BOLD signal decreased significantly when perceptual fading occurred and increased when the stimulus was seen again. The BOLD signal response in V2d and V3v is similar to that of V1v, but only reaches significance for the downward trend following a transition to the “no see” state. The BOLD signal does not modulate in V3d. (b) (c) and (d): The BOLD signal change averaged across voxels within subjects' ROIs (whole retinotopic areas) and across hemispheres. (b) When the stimulus was presented contralaterally to V1v and directly within V1v's response field, the BOLD signal decreased when perceptual fading occurred and increased when the stimulus was seen again. (c) The same result was observed when the stimulus was presented contralaterally but outside of V1v's response field. (d) The same result was observed when the stimulus was presented ipsilaterally. The BOLD signal response in V1d, V2v, and V3v is similar to that of V1v. The BOLD signal does not modulate in V2d and V3d. Statistics (N = 6): those areas that are significantly different than 0 in the two-tailed t-test (p<0.05) are marked as “*,” and marked as “***” for the two-tailed z-test (p<0.05).

Event-related average timecourses were computed within retinotopic cortical regions of interest (ROIs) *directly corresponding to the location of the target disk*. Timecourse segments representing the same perceptual state were averaged across runs (see methods). **Figure 3a** shows the average BOLD signal (averaged across 6 subjects) plotted in terms of percent signal change relative to the baseline defined by the level of the BOLD signal at the time of the transition as indicated by the button-press, set to the value zero here. When the target is presented directly to the specific ROI within V1v, V1d, and V2v, signal intensity rises after perceptual transitions from “no see” to “see” (*red*) and falls after transitions from “see” to “no see” (*blue*). Similar but weaker patterns were observed in the higher ventral (V3v) and dorsal (V2d and V3d) retinotopic areas. These data are consistent with a recent finding showing a reduction in activation in V1 during perceptual filling-in [16].

**Figure 4:**
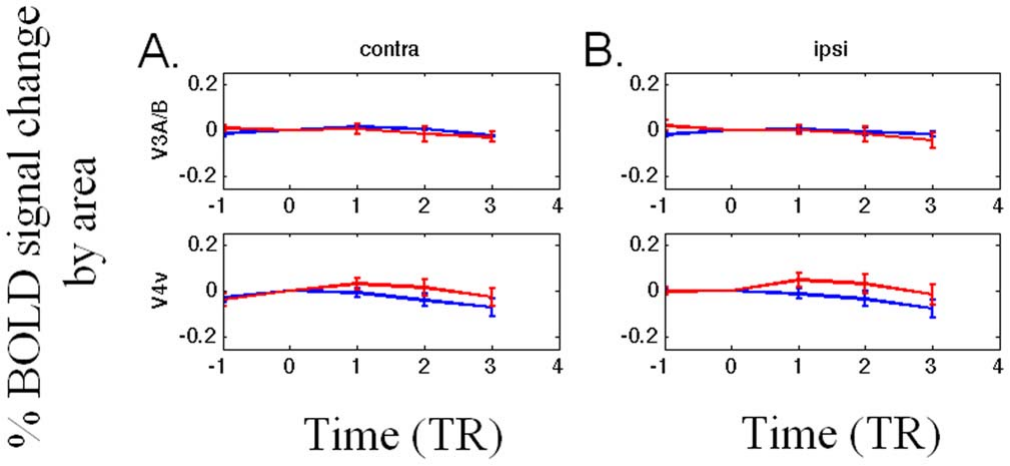
BOLD timecourses in V3A/B and V4v. The BOLD signal does not modulate in V3A/B and V4v. Statistics (N = 6): those areas that are significantly different than 0 in the two-tailed t-test (p<0.05) are marked as “*,” and marked as “***” for the two-tailed z-test (p<0.05).

In order to test whether perceptual fading is a local or global process, we also examined how BOLD signal varies with perceptual fading in retinotopic areas whose response fields do not correspond to the visual quadrant where the disk was located (**Figure 3**). If perceptual fading is a local process, the degree of BOLD signal change averaged across the whole retinotopic area should decrease relative to the ROI corresponding to the location of the perceptually fading figure, due to the increase in noise caused by averaging in voxels not involved in the filling-in process. Furthermore, no BOLD signal change should be observed when the target undergoing perceptual fading is located outside the visual area to which a retinotopic area responds. If perceptual fading is a global process, however, the opposite result should be observed. Our results in **Figure 3b** show that, when the target is presented contralaterally and directly located within the response field of V1v, signal intensity rises after perceptual transitions from “no see” to “see” (*red*) and falls after transitions from “see” to “no see” (*blue*) (**Figure 3b**). Similar results were observed when the target was presented contralaterally but outside the response field of V1v (**Figure 3c**). Note that an “ipsilateral effect” was observed, namely that the modulation occurred not only when the stimulus was presented contralaterally, but also when it was presented ipsilaterally (**Figure 3d**). Similar results were observed in V1d, V2v, and V3v. This effect is weaker in the dorsal retinotopic areas V2d and V3d (**Figure 3** **, bottom two rows**). In higher visual areas V3A/B and V4v (**Figure 4**), signal intensity does not modulate with perceptual transitions. Note that the present data are the first fMRI data reported for perceptual fading within ipsilateral as well as contralateral retinotopic cortex; The data described in [16] were only for cortex contralateral to the hemifield where the object actually faded; They wrote, however, “Our unilateral stimulus produced activation primarily in the contralateral (right) hemisphere, so we confined our analysis to that hemisphere.” Given the present results, we predict that similar activations would also be evident upon perceptual fading/filling-in if they reported corresponding retinotopic areas in the ipsilateral (in their case, left) hemisphere.

Similar results were observed when using a higher time resolution technique (see **Figure S1**). Instead of 16 slices per volume (TR  = 1600 ms), we used only 3 slices per volume (TR  = 300 ms) placed over the calcarine sulci in each subject to measure the BOLD signal response in bilateral V1 in three of the subjects from the previous experiment. We used this higher temporal resolution in order to explore the possibility that the contralateral areas might show a deviation from baseline before the ipsilateral areas, under the assumption that ipsilateral activation might be driven by feedback from contralateral areas (e.g. via the corpus callosum). **Figure S1** shows that the BOLD signal in V1 rises after perceptual transitions from “no see” to “see” and falls after transitions from “see” to “no see” with no noticeable temporal difference in deviations from baseline in the contralateral and ipsilateral cases. Importantly, however, the ipsilateral effect was observed here as well. These data essentially replicate the main effect reported here, but at a higher temporal resolution.

## Discussion

Our results show that BOLD signal modulates with perceptual transitions in certain retinotopic cortical areas whether or not the target disk is located in the quadrant corresponding to a retinotopic area's response field, suggesting that perceptual fading is not merely a local process, but is instead a global process. However, the exact mechanism of perceptual fading remains unknown. Possible mechanisms and confounds are discussed below.

### Microsaccades/Blinks Hypothesis

**Figure 5:**
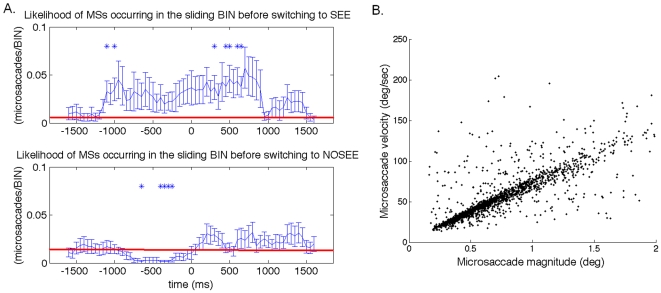
Rates of microsaccades around the time of a perceptual switch (time 0). (a) The rate of microsaccades rises before and after perceptual transitions from ‘no see’ to ‘see’, and decreases before perceptual transitions from ‘see’ to ‘no see.’ In the two-tailed simple t-test, those data points that are significantly different than the red baseline (defined as the mean of the first and the last data points across subjects) are marked as * (p<0.05). (b) Main sequence of all microsaccades (n = 1772 microsaccades).

It is possible that the BOLD signal rises in the “see” condition because of microsaccades or eyeblinks. Microsaccades/eyeblinks may directly or indirectly affect the BOLD signal. For example, the occurrence of a microsaccade/eyeblink may directly generate bilateral BOLD responses, as recently shown [17]. Or microsaccade/eyeblink occurrence may indirectly affect the BOLD signal, possibly by modulating global neuronal adaptation across the visual field during perceptual fading. It has been shown that the rates of both microsaccades and eyeblinks increase before perceptual switches to the ‘see’ state and decrease upon perceptual switches to the ‘no see’ state [18]–[23]. Replicating these past results, our eye-tracking data confirmed that the microsaccade rate correlates with the type of perceptual switch during perceptual fading (**Figure 5**). Our data show that the microsaccade rate was significantly greater than baseline both before and after a perceptual switch to the ‘see’ condition, and was significantly smaller than the baseline before a perceptual switch to the ‘no see’ condition. Therefore, this correlation suggests that the change of BOLD signal after perceptual switches could indeed be due to microsaccades.

However, while we cannot rule out that the ipsilateral activations observed here are due to microsaccades or eyeblinks, other data suggest that this is unlikely to be the case. In particular, the BOLD signal modulations due to microsaccades and eyeblinks are comparable in size in V2v versus V2d, and in V3v versus V3d [[17]; see **Figure S2** to see these data], yet here we find that the degree of BOLD signal modulation following a perceptual shift into or out of a perceptual ‘see’ or ‘no see’ state is only significant in V2v and V3v. Moreover, given that the baseline microsaccade rate is low (typically about one microsaccade per second [18]), and that the BOLD signal deflection from baseline that results from a microsaccade is on the order of 0.04% at its peak (see Figure S2), it is unlikely that small changes in the microsaccade rate can account for BOLD signal changes on the order of 0.2%, which is what we observe following perceptual fading bilaterally. Moreover, the BOLD signal deflection due to eyeblinks is comparably very large, on the order of 0.5% bilaterally (see figure 7 of reference 17). This suggests that the results we observe following the onset and offset of perceptual fading are not due to changes in eyeblink rate either. Finally, if our results are merely an artifact of microsaccade rate or eyeblinks, we would expect equivalent levels of activation in V2d and V2v, as well as in V3d and V3v, since this is what is observed in the event-related BOLD signal following either a microsaccade or an eyeblink. That we do not find similar activation in dorsal and ventral retinotopic cortex suggests that our present results are more likely to derive from processes that underlie perceptual fading, such as color mixing across the visual field [13]–[15], rather than a factor that is confounded with perceptual fading, such as microsaccade rate or eyeblinks.

However, because we cannot rule out the possible confound of microsaccade/eyeblink rate, it might seem that the present dataset should be recollected with an eyetracker that has a high enough temporal and spatial resolution to permit detection of microsaccades in the fMRI scanner. This, however, would not settle the matter, and it is difficult to imagine a method for presenting perceptual fading that can get around this problem, short of image stabilization upon the retina, which might itself eliminate the return to ‘see’ states entirely. Even if microsaccades could easily be detected in the scanner, the eye movements measured in the scanner would presumably demonstrate what we demonstrate here psychophysically by measuring eye movements during perceptual fading outside of the scanner; Namely, measuring eye movements in the scanner would presumably also show that the probability of microsaccade or eyeblink occurrence changes prior to and/or subsequent to the onset of perceptual fading. Thus the potential confound will remain even if eye movements are measured in the scanner.

### Ventral/Dorsal Asymmetry

Here we found significant modulation in BOLD signal with transitions in perceptual state from ‘see’ to ‘no see’ following perceptual fading, and vice versa. We have observed a similar asymmetry between ventral and dorsal retinotopic areas in the past. In particular, we carried out a similar study with motion induced blindness (MIB; n = 14 versus the present n = 6) [24], where subjects pressed a button indicating whether a dot had undergone MIB or not, and found that the BOLD signal response increases when the target dot, although continually present in the stimulus, subjectively reappears following MIB in V1v and V2v, but not corresponding dorsal areas. Interestingly, modulation of the BOLD signal occurred in corresponding ipsilateral areas in this study as well, although the physical target was only located in one quadrant of the visual field. There, as here, the ipsilateral BOLD signal that we observed could have arisen as a confounding influence of microsaccades or eyeblinks. Again, however, the fact that we observed solely ventral rather than ventral and dorsal retinotopic activation as a function of MIB onset/offset again suggests that the ipsilateral BOLD signal we observed was not due to this artifact (see **Figure S3**). And the previous arguments concerning the improbability of BOLD signals arising from microsaccades or eyeblinks accounting for the data observed in the case of perceptual fading also apply to the data acquired in the case of MIB.

In the data presented here, we observed greater differences in the BOLD signal between the ‘see’ and ‘no see’ states in ventral ROIs than in dorsal ROIs. This result in part replicates previous findings [25], [26] in which ventral visual areas V1, V2, Vp (identical to our V3v) and V4v showed greater activation during stimulus conditions in which image elements were perceptually grouped. The observation that ventral ROIs show greater differentiation between the two experimental conditions is potentially suggestive of a differential role in perceptual grouping between the “what” and “where/how” visual pathways [27], [28]. However, there is, to our knowledge, no neurophysiological evidence linking functional specialization along the lines of ventral and dorsal processing streams and the ventral and dorsal regions of V2 and V3. All of the neurophysiological evidence that we are aware of suggests that the functional specifications of these regions are solely limited to differences in the quadrant of the visual field that they represent. However, recent neuroimaging studies using fMRI have reported asymmetries in ventral and dorsal activations in V1 and V2 [16] and V3 [25]. If such a functional asymmetry exists across these subregions, one might predict that they would lead to asymmetries in behavior between the upper and lower visual hemifields. There has been recent debate in the literature with respect to whether such hemifield-dependent behavioral asymmetries exist [29]–[36]; but see: [37], [38]).

It should be noted that even if there are not any differences in the local processing mechanisms of V2d and V2v, or V3d and V3v, there may be differences in the degree or type of feedback that the dorsal and ventral sub-regions receive. For example, there may be more attentional or other types of top-down feedback to the ventral sub-areas than to dorsal sub-areas. If feedback projections predominantly originate in the inferior temporal and occipitotemporal regions, it could be that V2v, V3v, and V4v receive more feedback than corresponding dorsal areas simply because of greater proximity.

Care must be taken when trying to create a link between ventral/dorsal pathways of visual processing and the ventral and dorsal regions of early retinotopic cortex. Asymmetries such as those found in the data reported here could manifest themselves for reasons having nothing to do with ventral/dorsal stream processing. For example, increased BOLD signal can arise due to an increase in the spatial extent over which neural activity occurs or through increases in the magnitude of neural activity that occurs within a fixed spatial extent. Asymmetries in either the spatial distribution or relative magnitude of activity between the ventral and dorsal regions of early visual cortex could account for the ventral/dorsal asymmetry reported here.

### Cortical Feedback Hypothesis

A cortical feedback account of why BOLD signal modulates with perceptual state in bilateral visual cortex during perceptual fading would argue that the ipsilateral effect contradicts a purely bottom-up account of perceptual fading. Because information about stimuli in a given visual hemifield is not sent to ipsilateral V1 [39], such ipsilateral BOLD signal changes in V1 can be inferred to arise from signals coming from the contralateral V1, corticothalamic connections, and/or from other, higher cortical areas.

These feedback signals might be involved in generating visual awareness. It is possible that neuronal activity in early visual areas will be activated by feedback signals whenever the target disk is consciously perceived. For example, it has been hypothesized that V1 participates in generating visual awareness by forming recurrent circuits with extrastriate areas [40]–[46] (but see [47], [48]).

Alternatively, cortical feedback does not have to be directly involved in generating visual awareness. For example, cortical feedback might be involved in attentional enhancement following the perceptual change [44], [49]. It is possible that, whenever the target disk is consciously perceived, neuronal activity in early visual areas will be activated by an attention-related feedback signal. Single-unit studies in the monkey have shown attentional modulation in V1 [50]–[53]. fMRI studies have also revealed strong attentional modulation effects in V1 [54]–[58]. Additionally, attentional modulation in V1 can also occur when subjects anticipate a visual stimulus without visual stimulation [59], which supports that V1 can be activated by visual imagery without visual stimulation [60].

To conclude, our results show that BOLD signal level following perceptual fading in early and ventral retinotopic areas decreases when an object subjectively disappears, and increases when the object reappears. This effect occurs whether the stimulus is presented contralaterally or ipsilaterally. While we have specified BOLD signal correlates of perceptual fading in early visual areas, future work will have to determine the exact mechanism underlying perceptual fading in these areas. One possibility is that the non-local effects of perceptual fading result from non-local operations such as the feature mixing hypothesized to take place across the visual field upon perceptual fading [13]–[15]. Another possibility is that the non-local effects of perceptual fading reported here result from feedback. Another possibility is that they result from confounding effects of microsaccades and/or eyeblinks. Future work will have to tease apart which, if any, of these three possible explanations of the ipsilateral BOLD signal effects observed upon perceptual fading is the correct account.

## Materials and Methods

### Subjects

Six volunteers (of both genders between the ages of 18 and 41, including one author) were run in the first fMRI experiment. Three of the six subjects took part in the second fMRI experiment. All had normal depth perception and normal or corrected-to-normal visual acuity. Subjects were paid twenty dollars per fMRI session. The study conformed to the Code of Ethics of the World Medical Association (Declaration of Helsinki) and was approved by the Dartmouth committee for the Protection of Human Subjects and Dartmouth's internal review board. All participants gave written informed consent.

### Experimental Design

Each subject (N = 6) participated in an average of 8.3 runs in the scanner (range  = 7∼11). Each run lasted 403.2 sec (252 TRs; 1 TR  = 1600 ms), and in each run, there were 4 stimulation blocks (76.8 sec each), interleaved with 5 blank periods (19.2 sec each) (**Figure 1b**). In each stimulation block, an individually equated equiluminant green disk was presented in one of the four quadrants (left top, left bottom, right top, and right bottom) on a dark orange background that was the same for all subjects. The order of the 4 stimulation blocks, each containing a green disk in one and only one of the four quadrants, was randomized without replacement for each run. Stimuli were projected from a digital data projector (refresh rate 60 Hz) onto a plexiglass screen outside the bore of the magnet, and viewed via a tangent mirror inside the magnet that permitted a maximum of 22°×16° visible area. The projected image was smaller than this and subtended approximately 17°×12°. The fixation spot was a small square subtending 0.25° of visual angle, which changed color between blue/yellow (CIE, x = 0.151, y = 0.103) and red/green (CIE, x = 0.628, y = 0.341) every 3.21 s on average. The background was always orange (CIE: x = 0.454, y = 0.469), even during the blank period.

In order to optimize perceptual fading, the luminance of the green disk was adjusted to be subjectively equiluminant to that of the orange background for each subject independently using the minimal flicker technique [61]. Before the experiment, we presented a green (CIE: x = 0.315, y = 0.577) flashing (30 Hz) square that subtended 1.5° visual angle in the center of an orange background (CIE: x = 0.454, y = 0.469) and let the subjects adjust the green component of the square's color until minimal subjective flicker was reported. The color of the square was then fixed and applied to the green disk for the recording sessions. We also blurred the contour of the green disk by linearly transitioning between the color of the disk and the color of the background. The center of the green disk had a diameter subtending 1.5°, surrounded by a linear gradient between green and the background color that brought the overall blurred disk diameter to 2.25° visual angle. The disk was centered 4.25° (left or right from vertical midline) and 1.75° (above or below horizontal midline) depending on the quadrant in which it was located.

Subjects reported their current perceptual state by pressing a button with their right hand. They were asked to press this button with their right index finger when they did not see the green disk even if they knew it to be there, and release the button when they did see it.

Eye movements, wakefulness, and attention to the fixation point were controlled for by requiring subjects to report whether the fixation had changed color by pressing another button with their left index finger. The fixation point was 0.2°×0.2°, located at the center of the screen. The fixation point changed color randomly from blue/yellow to red/green on average every 3.1 seconds. This color change occurred an equal number of times during each block and the same number of times in each run.

A second, experiment was conducted on three of the six subjects who took part in the main experiment. All of the stimulus procedures were identical in this experiment; however, the acquisition of fMRI data was performed by placing only three slices bilaterally over the center of the calcarine sulcus. As a result, the TR was 300 ms in this experiment, providing better time resolution of the activations within area V1. Otherwise all aspects of the experiment were identical.

### MRI Scans

Anatomical and functional whole-brain imaging was performed on a 1.5 T GE Signa scanner using a standard head coil. T1-weighted anatomical images were acquired using a high-resolution 3-D spoiled gradient recovery sequence (SPGR; 124 sagittal slices, TE  = 6 ms, TR  = 16 ms, flip angle  = 25°, 1×1×1.2 mm voxels, FOV = 240×240×256 mm^3^). Functional images were collected using a gradient spin-echo, echo-planar sequence sensitive to blood-oxygen level-dependent contrast (T2*) (16 slices per volume (3 slices in experiment 2), 3.75 mm in-plane resolution, 4.5 mm thickness, 1-mm skip, TR  = 1600 ms (300 ms in experiment 2), Echo time  = 35 ms, flip angle  = 90°).

### fMRI Data Analysis

Data were analyzed offline using BRAIN VOYAGER (BV) 4.9.6 and MATLAB software developed in house. Effects of small head movements were removed using BV's motion correction algorithm. Functional data were not smoothed in the space domain. Oscillations in the time course with period greater than or equal to 3 times the length of a condition block were removed.

### Retinotopic Mapping

Retinotopy was carried out on all subjects (N = 6) using standard phase-encoding techniques (4.5 mm thickness and 3.75-by-3.75 mm in-plane voxel resolution, inter-slice distance 1 mm, TR  = 1600 msec, flip angle  = 90°, field-of-view  = 240×240×256 mm, interleaved slice acquisition, matrix size = 64×64; 16 slices oriented along the calcarine sulcus) with the modification that two wedges of an 8 Hz flicker black and white polar checkerboard grating were bilaterally opposite (like a bowtie), to enhance signal to noise [62], [63]. Wedges occupied a given location for 2 TRs (3.2 seconds) before moving to the adjacent location in a clockwise direction. Each wedge subtended 18 degrees of 360 degrees. 9.6 seconds (6 TRs of dummy scans) were discarded before each run to bring spins to baseline. 168 volumes were collected on each run. A minimum of 7 wedge runs were collected for each subject and then averaged to minimize noise before retinotopic data analysis in BV 4.9.6. A minimum of three runs were collected per subject using expanding 8 Hz flickering concentric rings that each spanned approximately one degree of visual angle in ring width. Each ring was updated after one TR (1.6 s) after which it was replaced by its outward neighbor, except that the outermost ring was replaced by the innermost ring, whereupon the cycle was repeated. Retinotopic areas, including V1, V2d, V2v, V3d, V3v, V4v/VO1, and V3A/B, were defined as masks on the basis of standard criteria [62], assuming a contralateral quadrant representation for V2d, V2v, V3d, and V3v, and a contralateral hemifield representation for V1, V4v/VO1, and V3A/B [64]. V4v and the hemifield representation just anterior to it, called VO1 [65] were combined into a common mask because the border between these regions was not distinct in all subjects, as was true for the combination of V3A and V3B into a common V3A/B mask.

We further identified ROIs directly corresponding to the size/location of the target disk, one for each quadrant, within retinotopic areas V1v, V1d, V2v, V2d, V3v, and V3d. These specific ROIs were defined by identifying the eccentricity and the polar angle of the target disk on the eccentricity mask and the polar mask respectively, and then taking the intersection of these two masks.

### fMRI Timecourse Data Analysis

Because our goal was to compare the BOLD signal after perceptual switches, we only averaged data from ‘see’ conditions that followed ‘no see’ conditions, and vice versa. Thus the first button-press in each stimulation block, which always corresponded to a ‘see’ state (induced by the stimulus onset at the beginning of each stimulation block at the end of a ‘no disk’ epoch), was excluded to eliminate nonspecific onset effects that possibly had nothing to do with the reappearance of the vanished disk after a ‘no see’ state. Also, if the last button-press happened within 3 TRs before the end of a stimulation block, it was excluded to eliminate any possible offset effects.

In areas V1v, V1d, V2v, V2d, V3v, and V3d, we further show the timecourse for the following three conditions, based on whether the target dot was presented inside (labeled “contra within”), outside (labeled “contra outside”), or ipsilaterally (labeled “ipsi”) to a ROI's corresponding quadrant visual field. In areas V3A/B and V4v, we only compared the timecourse based on whether the target dot was presented contralaterally (the “contra” condition) or ipsilaterally (the “ipsi” condition) to a ROI because response fields within these areas are known to be large, and thus may cross the horizontal meridian if not the vertical meridian.

A two-tailed t-test was carried out to compare the means of TR = 0 to TR = 1, TR = 2, and TR = 3 individually. A two-tailed z-test was carried out to compute the z score using the formula z = (ΣT_j_)/√{Σ (F_j_/(F_j_−2))}, in which T_j_ equals the t scores (of TR = 1, TR = 2, and TR = 3) and the F_j_ equals their degrees of freedom. This z-test reaches significance if several data points are collectively significantly different from zero, even though each individual data point might not be, as measured by a t-test.

A potential problem with timecourse averaging such as that used here is that variable durations of perceptual states tend to blur the later part of the time course of individual responses, because the probability of switching into the opposite perceptual state increased with time. In order to avoid the possible contamination that would arise by averaging in BOLD signal arising from switches into the opposite perceptual state, we excluded those percepts shorter than 2TRs and limited the analysis interval to the first 3TRs after the button-press in experiment 1. This allowed us to be certain that subjects' percepts were 100% in either the ‘see’ or ‘no see’ states within the first 3 TRs after a perceptual switch. Thus BOLD signal averages shown are due to ‘pure’ perceptual states following a perceptual transition from a ‘see’ to ‘no see’ state, or vice versa. In experiment 2 more very short duration TRs were analyzed, in order to correspond to the durations considered in analyzing experiment 1.

### Eye-Tracking Experiment and Microsaccade Data Analysis

We repeated the exact experiment with eyetracking outside the scanner on ten subjects (four of them had participated in the fMRI experiment). All the stimuli and procedures were identical except that there was no baseline (no disk) condition between ‘disk on’ stimulation blocks, and subjects were sitting rather than lying on their backs. The screen, disk, and cross elements subtended the same visual extent as in the fMRI experiment. Eye movements were recorded using a SRresearch Eyelink2 system for the left eye. Eye position was sampled at 250 Hz in the left eye. Observers ran in one or two sessions, each equivalent to a run of the fMRI experiment. Observers were required to maintain fixation on each trial. A miniature video camera, attached to an adjustable headband and bar, was fitted about 2 cm below the subject's left eye, and eye movements were calibrated to a disk that moved to nine positions on the screen in random order. Observers rested their chin in a stable rest. The distance from their eyes to the screen was adjusted such that the visual angles of the stimuli were the same as that in the scanner. The head was not otherwise constrained, although observers were instructed to maintain their head perfectly still. Small head movements could be discounted online by the eye-tracker software using the output of four cameras mounted on the monitor.

Linear drift in eye traces in both x-channel and y-channel (typically due to sliding of the headband down subject's foreheads) was corrected before data analysis. We took the total amount of drift within a run and divided that amount by the total duration of a run. This ‘drifting per unit of time’ was then corrected for each time point. Eyeblinks and large eye movements were then identified by the algorithm of Engbert and Kliegl [66] (velocity threshold  = 10 std.; minimum duration  = 5 units of time; velocity type  = 2; amplitude bigger than 3.33 visual degrees). Data in a time window starting 400 msec before and ending 600 msec after each eyeblink or large eye movement were not used in the following analysis. Microsaccades were then located using the same algorithm [66] (velocity threshold  = 10 std.; minimum duration  = 4 units of time; velocity type  = 2; Note that we used a higher velocity threshold than what was used in [67] to identify eyeblinks and large eye movements. After removing these eyeblinks and large eye movements, we applied the same algorithm again to identify microsaccades). A secondary screening procedure was used to exclude those detected microsaccades that were smaller than 0.15 visual degrees and larger than 2 visual degrees. If there were any two microsaccades (or a cluster of microsaccades) identified by the above algorithm that had an interval shorter than 80 ms between them, only the one with the largest amplitude contributed to the final analysis. This was done because microsaccades are followed by a refractory period during which microsaccades do not occur [67].

Each subject's button press time points were identified as the onset of the perceptual switches, which correspond to the 0 points in **Figures 3** **,** **4**, and **5**. Within each subject, microsaccade/eyeblink ‘rate’ was plotted by calculating the total number of microsaccades/eyeblinks within a 50 ms window around each data point. The interval between each data point is 50 ms. The value of each data point was then recalculated by taking the mean of the five data points around it. Error bars indicate standard error of the mean rate across subjects. A two-tailed paired t-test was carried out to test whether there was a difference in microsaccade/eyeblink rate from the baseline. Those data points that are significantly different than the baseline are marked as * (p<0.05). The baseline is defined by the mean of the first and last data point.

## Supporting Information

Figure S1 Higher time resolution BOLD timecourses. When the stimulus was presented contralaterally to V1 (n = 3), the BOLD signal decreased when perceptual fading occurred and increased when the stimulus was seen again (left column). The same result was observed when the stimulus was presented ipsilaterally to these areas (right column). The BOLD signal change was averaged across voxels within three subjects' V1 and across hemispheres. One TR (300 ms) represents the acquisition time for one 3-slice volume placed along the calcarine sulcus.
(1.31 MB TIF)

Figure S2 Bold signal following a microsaccade. Bold signal following a microsaccade is comparable in dorsal and ventral retinotopic areas. The data here are from [reference 17, compare Figure 7a, n = 3], and show event-related BOLD signal following a microsaccade in V1, V2d, V2v, V3d, and V3v. For details on methods, see [17].
(0.83 MB TIF)

Figure S3 The differences of BOLD timecourses (TR  = 1.6 seconds) upon perceptual switches in V1v, V1d, V2v, V2d, V3v, and V3d for the onset and offset of motion-induced blindness (MIB). Note that the same basic pattern of data is apparent following both perceptual fading and MIB. The data here are reproduced from reference 24, Figure 4, for purposes of comparison with the present data concerning the BOLD signal changes associated with perceptual fading. The BOLD signal change averaged across voxels within subjects' ROIs and across hemispheres relative to the 16 slice volume acquisition (TR)  = 0 position, corresponding to the beginning of a volume in which the subject reported a perceptual switch. The area is marked ‘contra within’ when the target was located inside the corresponding visual field, and marked ‘contra outside’ when target was located on the contralateral side to the ROI but outside the corresponding visual quadrant. The area is marked ‘ipsi’ when the ROI was on the same side as the target that underwent MIB. The x-axis shows the time in units of TR (1.6 seconds), and the y-axis shows the percentage change of BOLD signal (%). The results show that the BOLD signal increased when the stimulus reappeared from MIB in V1v and V2v. The same result was observed when the stimulus was presented ipsilaterally to these areas. Statistics: N = 14; A two-tailed t-test was carried out to compare the value of TR = 0 (set to be zero) to the means of each TR individually. Those data points that are significantly different than 0 are marked as ‘*’ (p<0.05). For details on methods, see [24].
(0.06 MB TIF)
