# Supplementary figures and images for: BOLD Signal in Both Ipsilateral and Contralateral Retinotopic Cortex Modulates with Perceptual Fading
Source: PLoS One. 2010 Mar 11;5(3):e9638. doi: 10.1371/journal.pone.0009638 (PMC2836375; doi:10.1371/journal.pone.0009638)

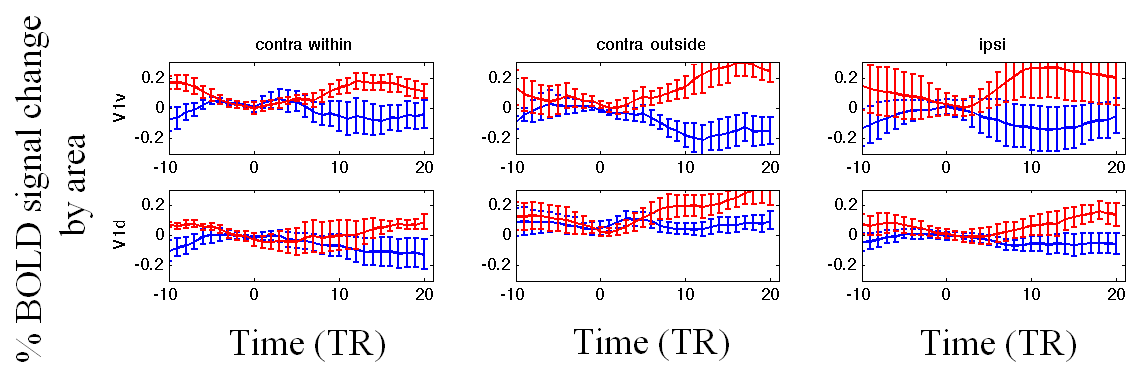

Supplement: Figure S1 — Higher time resolution BOLD timecourses. When the stimulus was presented contralaterally to V1 (n = 3), the BOLD signal decreased when perceptual fading occurred and increased when the stimulus was seen again (left column). The same result was observed when the stimulus was presented ipsilaterally to these areas (right column). The BOLD signal change was averaged across voxels within three subjects' V1 and across hemispheres. One TR (300 ms) represents the acquisition time for one 3-slice volume placed along the calcarine sulcus. (1.31 MB TIF) [file pone.0009638.s001.tif]

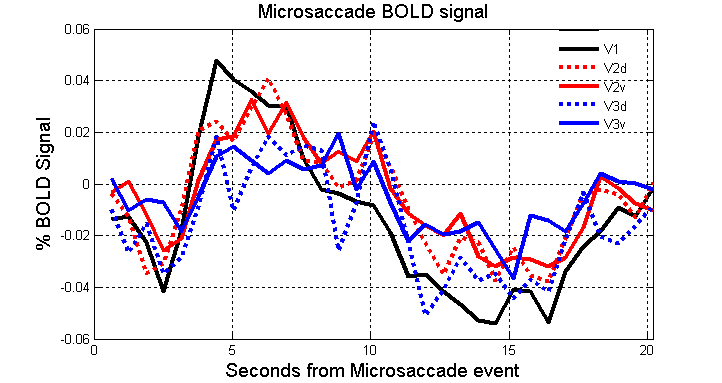

Supplement: Figure S2 — Bold signal following a microsaccade. Bold signal following a microsaccade is comparable in dorsal and ventral retinotopic areas. The data here are from [reference 17, compare Figure 7a, n = 3], and show event-related BOLD signal following a microsaccade in V1, V2d, V2v, V3d, and V3v. For details on methods, see [17]. (0.83 MB TIF) [file pone.0009638.s002.tif]

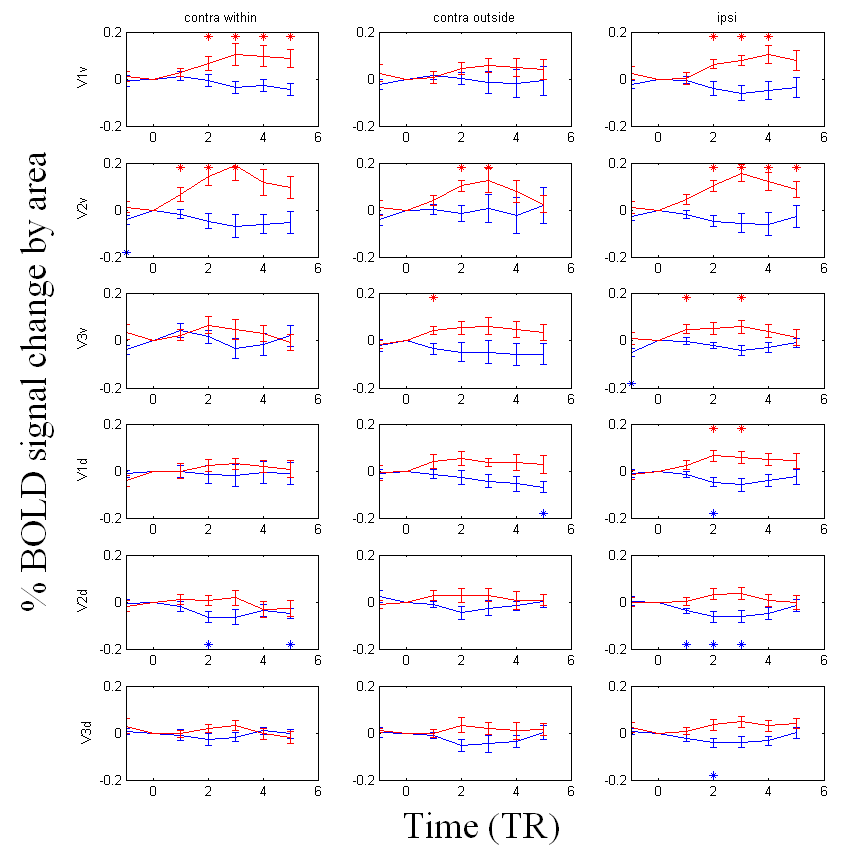

Supplement: Figure S3 — The differences of BOLD timecourses (TR = 1.6 seconds) upon perceptual switches in V1v, V1d, V2v, V2d, V3v, and V3d for the onset and offset of motion-induced blindness (MIB). Note that the same basic pattern of data is apparent following both perceptual fading and MIB. The data here are reproduced from reference 24, Figure 4, for purposes of comparison with the present data concerning the BOLD signal changes associated with perceptual fading. The BOLD signal change averaged across voxels within subjects' ROIs and across hemispheres relative to the 16 slice volume acquisition (TR) = 0 position, corresponding to the beginning of a volume in which the subject reported a perceptual switch. The area is marked ‘contra within’ when the target was located inside the corresponding visual field, and marked ‘contra outside’ when target was located on the contralateral side to the ROI but outside the corresponding visual quadrant. The area is marked ‘ipsi’ when the ROI was on the same side as the target that underwent MIB. The x-axis shows the time in units of TR (1.6 seconds), and the y-axis shows the percentage change of BOLD signal (%). The results show that the BOLD signal increased when the stimulus reappeared from MIB in V1v and V2v. The same result was observed when the stimulus was presented ipsilaterally to these areas. Statistics: N = 14; A two-tailed t-test was carried out to compare the value of TR = 0 (set to be zero) to the means of each TR individually. Those data points that are significantly different than 0 are marked as ‘*’ (p<0.05). For details on methods, see [24]. (0.06 MB TIF) [file pone.0009638.s003.tif]
